# Prp19/CDC5L promotes gastric cancer via activation of the MAPK pathway-mediated homologous recombination

**DOI:** 10.7150/ijbs.101962

**Published:** 2025-01-27

**Authors:** Shengkui Qiu, Feiran Wang, Xuesong Gao, Weiyu Guan, Ting Dai, Lei Yin, Fei Wang, Jinjie Sun, Peng Guo, Hao Wu, Shichun Feng, Chong Tang

**Affiliations:** 1 Department of General Surgery, Nantong First People's Hospital, Affiliated Hospital 2 of Nantong University, Nantong 226001, Jiangsu Province, China.; 2 Department of General Surgery, Affiliated Hospital of Nantong University, Nantong 226000, Jiangsu Province, China.; 3 Department of Gastroenterology, Nantong First People's Hospital, Affiliated Hospital 2 of Nantong University, Nantong 226001, Jiangsu Province, China.; 4 Nantong Clinical Medical College, Kangda College of Nanjing Medical University, Nantong 226001, Jiangsu Province, China.

**Keywords:** gastric cancer, Cell division cycle 5-like protein, homologous recombination, MAPK pathway

## Abstract

**Background:** Recent advances in gastric cancer (GC) treatment have not substantially improved the 5-year survival rate nor have they significantly reduced the high recurrence rate. This highlights the need for further research to explore the underlying mechanisms of GC. Cell Division Cycle 5-Like Protein (CDC5L) has been implicated in various malignant behaviors of tumors.

**Methods:** We investigated the expression of CDC5L in gastric cancer (GC) using data from The Cancer Genome Atlas (TCGA) and clinical specimens. To explore the role of CDC5L in GC, we conducted *in vitro* and *in vivo* assays, alongside molecular mechanism studies using luciferase reporter assays, co-immunoprecipitation (CO-IP), and mass spectrometry (MS).

**Results:** Our findings indicate a significant elevation of CDC5L in GC, with CDC5L overexpression correlating with poorer survival outcomes, advanced TNM stages, and higher pathological grades in GC patients. *In vitro*, interference of CDC5L markedly inhibited GC progression. We discovered that the Pre-mRNA Processing Factor 19 (Prp19) directly binds to the CDC5L promoter, enhancing its transcription and inhibiting its lysosome-mediated degradation. Additionally, CO-IP and MS assays revealed that CDC5L interacts with MAPK1, activating the MAPK signaling axis and consequently augmenting homologous recombination in GC.

**Conclusions:** In summary, our study confirms that Prp19 upregulates CDC5L expression, which binds to MAPK1, thereby promoting GC progression via the MAPK pathway-mediated homologous recombination. Targeting CDC5L could be a promising strategy in the precision therapy of GC.

## 1. Introduction

Gastric cancer (GC) ranks as the fifth most prevalent cancer and the fifth leading cause of cancer-related mortality worldwide 1. Despite advancements in treatment, the prognosis for GC remains grim, primarily due to tumor invasion and metastasis. The 5-year survival rate for GC is below 20% 2, 3, underscoring the urgent need to elucidate the molecular mechanisms underlying tumor progression to improve GC treatment.

Cell division cycle 5-like protein (CDC5L), a crucial component of the human Prp19/CDC5L complex, plays a vital role in pre-mRNA splicing 4-6. Prp19/CDC5L is implicated in cell cycle regulation, mitotic checkpoint control, and DNA repair 7-10, linking it to cancer pathogenesis, as evidenced in non-small cell lung cancer, hepatocellular carcinoma, and colorectal cancer 11-14. The specific function of Prp19/CDC5L in GC progression, however, remains to be thoroughly explored.

DNA double-strand breaks (DSBs), the most severe form of DNA damage, are the main mechanism by which chemotherapy drugs, especially anthracyclines and platinum drugs, exert cytotoxicity 15-17. DNA double-strand breaks repair (DSBR) includes two main pathways, non-homologous end-joining (NHEJ) and homologous recombination (HR) 18-20, with HR being an error-free method 21, 22. Oxaliplatin-based chemotherapy, recommended for advanced GC, faces the significant challenge of chemoresistance 23-25. Oxaliplatin induces DSBs by forming platinum-DNA adducts, targeting rapidly dividing cells 26-28. Emerging evidence suggested that HR played a key role in promoting chemoresistance 29, 30, but the precise molecular mechanisms remain elusive. The mitogen-activated protein (MAP) kinase signaling cascade, specifically the Ras-Raf-MEK-MAPK axis, is an evolutionarily conserved pathway critical in regulating cell growth, proliferation, and differentiation through a series of phosphorylation reactions 31, 32. This pathway is implicated in various cancers, including GC and melanoma 33-35, and is associated with malignant behaviors like drug resistance and DNA damage repair 36, 37. Yet, the potential regulatory role of CDC5L in the MAPK pathway and its contribution to GC progression is not well understood.

Our study reveals that CDC5L is upregulated in GC, promoting tumor proliferation, migration, invasion, and oxaliplatin resistance. We demonstrate that Prp19 binds with CDC5L to enhance its expression and that CDC5L interacts with MAPK1, thereby activating the MAPK pathway. These findings position CDC5L as a promising therapeutic and prognostic target in GC.

## 2. Methods

### 2.1 Patients and specimens

All GC tissues and adjacent normal tissues were acquired from patients at Nantong First People's Hospital from January 2016 to January 2019. Patients receiving chemotherapy and radiotherapy were excluded. The fresh tissues were immediately frozen and stored in the liquid nitrogen. The clinical diagnosis of the patients was determined by professional pathological analysis. Written informed consent was obtained from all patients involved and the study has been approved by the Ethics Committee of the Nantong First People's Hospital.

### 2.2 Cell culture

Cell lines were acquired from the Shanghai Institutes for Biological Sciences and maintained in RPMI 1640 medium supplemented with 10% fetal bovine serum (Invitrogen, USA) and 1% penicillin/streptomycin (Gibco, USA), in an atmosphere containing 5% CO2 at 37 ℃.

### 2.3 Transfection and plasmid construction

ShRNAs and plasmids designed and purchased from GenePharma (China) were packaged in lentivirus vectors. Transfection procedures were performed using Lipofectamine 3000 (Invitrogen, China), following the manufacturer's protocol.

### 2.4 RNA isolation and quantitative real time PCR (qRT-PCR)

RNA isolation was achieved using TRIzol reagent (Invitrogen, USA), and reverse transcription was conducted with Takara RT reagent (Takara Bio, Japan). The qRT-PCR was executed on an ABI PRISM 7300 Sequence Detection system (Applied Biosystems, USA), employing SYBR Premix Ex Taq™ II (Takara, China), with the 2^- △△ct^ method applied for relative expression analysis. Primer sequences are provided in Supplementary Table 1.

### 2.5 Western blot (WB) and immunoprecipitation (IP)

For protein analyses, extraction was performed using RIPA buffer (Beyotime, China), followed by separation via 10% SDS-PAGE (Beyotime, China) and transfer to nitrocellulose membranes. Proteins were incubated with primary antibodies overnight at 4 ℃, followed by secondary antibodies for 2 h at 37 ℃, and visualized using ECL chemiluminescence reagent. Details of antibodies are in Supplementary Table 2.

Immunoprecipitation involved pre-incubating protein A/G-agarose beads (Santa Cruz Biotechnology, USA) with antibodies for 4 h at 4 ℃, followed by overnight incubation with proteins at the same temperature. After boiling in SDS for 5 min at 95 ℃, the immunoprecipitated proteins were analyzed via Western blot and mass spectrometry (MS) (Thermo Scientific, USA).

### 2.6 CCK-8 assay

GC cells were seeded in 96-well plates at a seeding density of 5000 cells per well. CCK-8 reagent (MCE, China) was applied to the wells, and absorbance readings at 450 nm were subsequently taken to assess cell viability.

### 2.7 Colony formation assay

GC cells were evenly distributed in 6-well plates at 1000 cells per well and cultured for a fortnight. Post-staining, colony images were captured for analysis.

### 2.8 5-Ethynyl-2′-deoxyuridine (EdU) assay

For assessing DNA synthesis, GC cells were placed in 96-well plates at a density of 10,000 cells per well. Following incubation with EdU solution (Beyotime, China) for 2 hours, cells were fixed with 4% paraformaldehyde. Reaction mixture was added as per the protocol, and nuclei were counterstained with Hoechst 33342. Fluorescence microscopy (Nikon, Japan) was employed for image acquisition.

### 2.9 Cell cycle and apoptosis analysis

GC cells were harvested, ethanol-fixed, and processed for cell cycle analysis following Beyotime protocol (China), with data acquired via flow cytometry (Beckman, USA). For apoptosis detection, cells were treated with PE-Annexin V Apoptosis Detection Kit (BD Biosciences, USA) and analyzed using flow cytometry.

### 2.10 Tranwell assay

Transwell inserts (Millipore, USA) were placed in 24-well plates. The bottom chamber was filled with 600μl RPMI 1640 containing 10% FBS, while 20,000 cells in 200μl of RPMI 1640 were seeded into upper chambers. After 48 hours, cells were fixed with methanol, stained with crystal violet, and imaged using an inverted microscope (Nikon, Japan).

### 2.11 Wound-healing assay

GC cells were seeded in the 6-well pates. To avoid the impact of proliferation on the scratch assay, mitomycin (1ug/ml) was added in the medium. A sterile pipette tip was used to create thin scratches of constant width along the center of each hole after the cells had adhered to the bottom of the plate. The images were photographed using an inverted microscope (Olympus, Japan).

### 2.12 Dual‑luciferase reporter assay

The CDC5L target sequence was subcloned into the pGL3-basic vector and the Prp19 target sequence was transferred into the pEGFP-N1 vector. GC cells were seeded in the 24-well plates and transfected with the luciferase vector. The efficiency of transfection was reflected by Firefly luciferase activity normalized to Renilla luciferase activity.

### 2.13 Immunofluorescence (IF) assay

GC cells, seeded on 35 mm culture plates, were cultured overnight. Fixation was done using 4% paraformaldehyde, followed by permeabilization with 0.1% Triton X-100. Primary antibody incubation occurred at 4 ℃ overnight, succeeded by a 2-hour room temperature incubation with secondary antibodies in darkness. Nuclei staining was achieved using DAPI, and a Leica SP5 confocal microscope system (Leica Microsystems, Germany) was employed for image acquisition. Antibody details are provided in Supplementary Table 2.

### 2.14 Immunohistochemical (IHC) analysis

Paraffin-embedded GC tissues underwent baking, deparaffinization, and rehydration. Peroxidase activity suppression and nonspecific staining blocking were performed. Antibody incubation was carried out at 4 ℃ overnight, followed by 1-hour incubation with corresponding secondary antibodies at 37 ℃. IHC scoring combined frequency and intensity: frequency scores ranged from 0 (< 5% positive cells) to 4 (≥ 75%), and intensity scores ranged from 0 (negative), 1(weak), 2 (medium) to 3 (strong). Antibody details are in Supplementary Table 2.

### 2.15 Neutral comet assay

Neutral comet assay was conducted by using the Comet Assay Kit (R&D Systems, USA) via the manufacturer's specifications. Cells were digested at a density of 100000 cells/mL and mixed with molten LMAgarose at a ratio of 1:10. Then the cells were immobilized on comet slides. Lysis was performed for 1 h followed by electrophoresis at 4 ℃ for 45 min. Cells were finally stained with the SYBR Gold (Invitrogen, USA) and analyzed with a fluorescence microscope (Nikon, Japan).

### 2.16 Organoid model

GC tissues were cropped and ground into pieces and then digested by collagenase A. Cells were resuspended in Matrigel (R&D Systems, USA) adding growth factors. The mixture was then seeded in 24-well plates and cultured with Organoid Growth Medium (StemCell Technologies, Canada). The organoid growth was photographed daily.

### 2.17 *In vivo* tumorigenesis model

The 4-week-old female BALB/C were purchased from Beijing Vital River Laboratory Animal Technology Co., Ltd and the animal experiments were approved by the Ethics Committee of the Nantong First People's Hospital. Treated GC cells were resuspended at the concentration of 1 × 10^6^ GC cells and were then injected into the groin area of nude mice. The tumor volume and growth curve were observed and measured. After 4 weeks, the mice were sacrificed. Afterwards, the tumor volume and size were measured.

### 2.18 Statistical analysis

Data of each experiment in this study were shown as means ± SD. P< 0.05 was considered as statistically significant. Chi-square test was applied to compare the count data. The differences between the groups were analyzed by independent t-test or one-way analysis of variance (ANOVA). Overall survival (OS) was plotted using the Kaplan-Meier method and was analyzed using the COX regression model. *P < 0.05, **P < 0.01, ***P < 0.001.

## 3. Results

### 3.1 The overexpression of CDC5L was positively correlated with poor prognosis in GC patients

**Figure 1:**
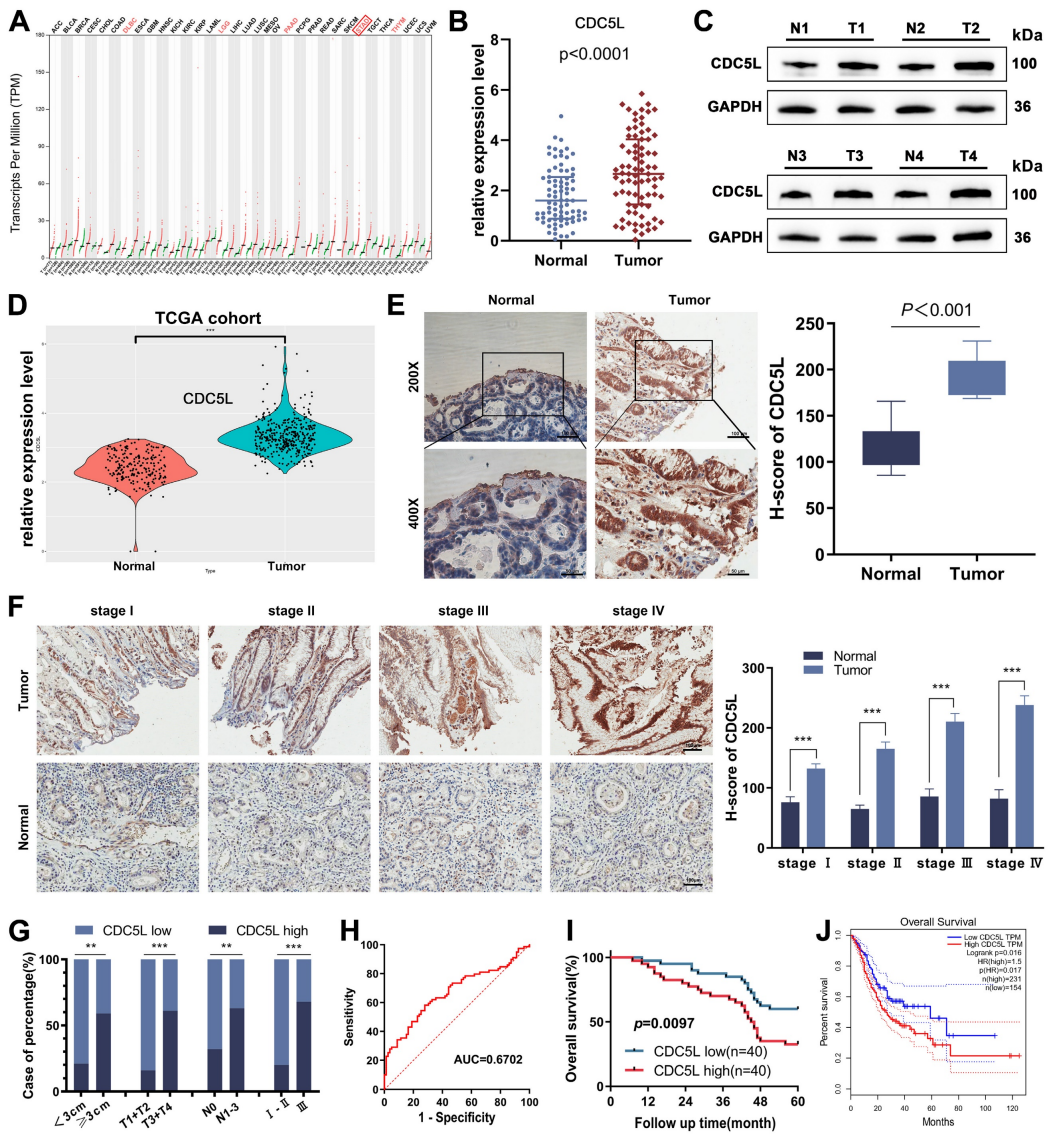
The overexpression of CDC5L was positively correlated with poor prognosis in GC patients. **A.** CDC5L was elevated in various cancer tissue compared with the normal tissue based on TCGA database. **B.** mRNA expressions of CDC5L in GC tissues and adjacent normal tissues were analyzed by qRT-PCR. **C.** Protein expressions of CDC5L in GC tissues and adjacent normal tissues were analyzed by WB. **D.** CDC5Lwas elevated in GC tissue compared with the normal tissue based on TCGA database. **E.** IHC was used to detect the expression of CDC5L in paired tumor tissues and adjacent normal tissues in GC patients. **F.** IHC was used to detect CDC5L expression levels in paired tumor and adjacent normal tissues stratified according to different pathological stages. **G.** Percentage of CDC5L expression in GC categorized by tumor size, T stage, lymph node metastasis, and TNM stage. **H.** The diagnostic value of CDC5L for GC was evaluated using ROC curves. **I.** The overall survival (OS) was evaluated by the Kaplan-Meier survival curve and the log-rank test from 80 GC patients according to CDC5L expression levels. **J.** The overall survival (OS) of GC patients from TCGA database was evaluated by the Kaplan-Meier survival curve and the log-rank test according to CDC5L expression levels. Error bars indicate SD. *P < 0.05, **P < 0.01, ***P < 0.001.

CDC5L's involvement in various malignant tumor behaviors prompted our investigation into its role in GC progression. Utilizing the GEPIA tool, we identified CDC5L upregulation in various tumors, including GC (Fig. 1A). We further confirmed this finding by analyzing CDC5L expression in GC and corresponding normal tissues using data from the TCGA database and our center's patients. In 80 GC patients from our center, both mRNA and protein levels of CDC5L were significantly higher in tumor tissues compared to normal tissues (Fig. 1B-C, Fig. S1A), a finding echoed by TCGA data (Fig. 1D). Immunohistochemistry (IHC) results also demonstrated elevated CDC5L expression in tumor tissues (Fig. 1E). Additionally, higher CDC5L levels were associated with advanced pathological stages (Fig. 1F). Analysis of baseline data from these 80 patients (Table 1) showed a positive correlation between CDC5L expression and larger tumor size, as well as advanced T and N stages (Fig. 1G). Moreover, CDC5L was identified as an independent prognostic factor for GC (Table 2 and Fig. 1H). Clinical data from our center confirmed that high CDC5L expression is linked to poor prognosis in GC patients (Fig. 1I), which was consistent with the survival data in TCGA (Fig. 1J). Collectively, these findings suggest that CDC5L upregulation contributes to the progression and poor prognosis of GC.

### 3.2 CDC5L promoted the proliferation of GC cells and inhibited cell cycle arrest in the G1 phase

**Figure 2:**
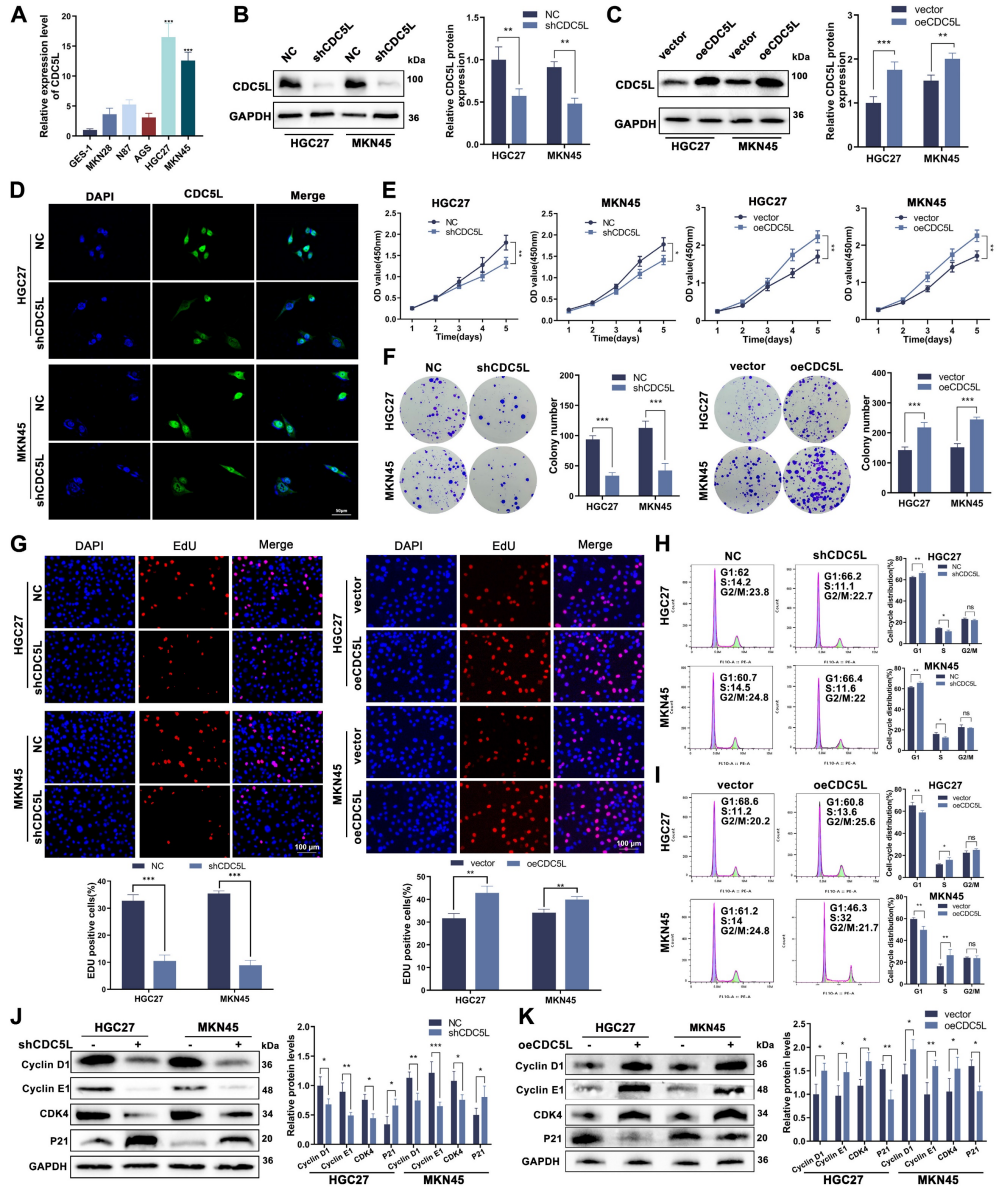
CDC5L promoted the proliferation of GC cells and inhibited cell cycle arrest in the G1 phase. **A.** QRT-PCR was utilized to detect the expression levels of CDC5L in GC cells. **B, C.** QRT-PCR and WB were used to detect the expression levels of CDC5L after silencing and overexpression in GC cells. **D.** IF was used for the detection of the localization and silencing efficiency of CDC5L in GC cells. **E-G.** CCK-8, Colony formation and EdU assays were used to detect the proliferation of GC cells after silencing and overexpression of CDC5L. **H, I.** Flow cytometry was used to detect the cell cycles of GC cells after silencing and overexpression of CDC5L. **J, K.** WB was used for the detection of expression levels of cell cycle related proteins after silencing and overexpression of CDC5L. Error bars indicate SD. *P < 0.05, **P < 0.01, ***P < 0.001.

In exploring the functional role of CDC5L, we observed its high expression in GC cell lines, particularly in HGC27 and MKN45, relative to GES-1, normal gastric epithelial cells (Fig. 2A, Fig. S1B). Subsequent experiments focused on these two cell lines. We developed CDC5L-silenced and -overexpressed models in HGC27 and MKN45, confirming transfection efficiency via western blot, qRT-PCR and immunofluorescence (Fig. 2B-D). Cell proliferation assays (CCK-8, colony formation, and EdU) demonstrated reduced proliferation upon CDC5L knockdown. When overexpressing CDC5L, the opposite conclusion was drawn (Fig. 2E-G). Cell cycle analysis revealed an increase in G1 phase arrest with CDC5L silencing, accompanied by decreased Cyclin D1, CDK4, and Cyclin E1 expression, and increased P21 expression. The opposite trend was obtained upon the overexpression of CDC5L (Fig. 2H-K). Taken together, our results indicated that CDC5L promotes GC cell proliferation and suppresses cell cycle arrest in the G1 phase of GC.

### 3.3 CDC5L inhibited the apoptosis and facilitated the invasion, migration, and EMT of GC cells

**Figure 3:**
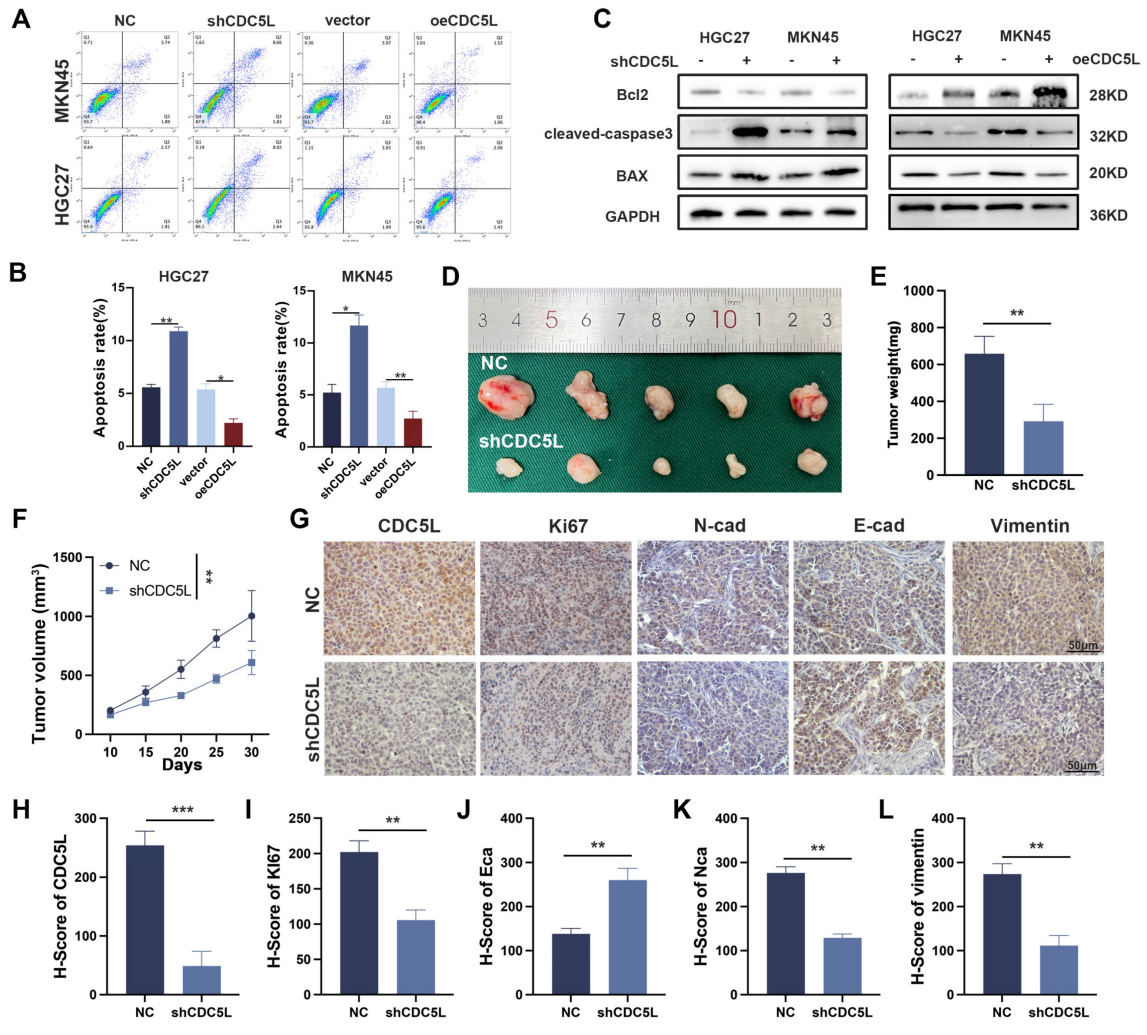
CDC5L inhibited the apoptosis and facilitated the EMT of GC cells. **A, B.** Flow cytometry was used to detect the apoptosis of GC cells after silencing and overexpression of CDC5L. **C.** WB was used for the detection of expression levels of apoptosis related proteins after silencing and overexpression of CDC5L. **D-F.** Subcutaneous tumorigenesis in nude mice was performed to evaluate the effect of CDC5L silencing on tumor proliferation and tumor volume and weight were measured. **G-L.** IHC was used to detect the expression of CDC5L, Ki67, E-cadherin, N- cadherin and Vimentin in nude mice. Error bars indicate SD. *P < 0.05, **P < 0.01, ***P < 0.001.

We investigated the role of CDC5L in GC cell invasion, migration, and epithelial-mesenchymal transition (EMT). Transwell and wound healing assays showed that CDC5L upregulation enhanced GC cell invasion and migration, whereas CDC5L silencing reduced these processes (Fig. S1C-F). Similarly, Western blot analysis indicated that CDC5L upregulation promoted EMT, a crucial process in GC progression (Fig. S1G), while its knockdown increased apoptosis rates of GC cells (Fig. 3A-B). Apart from this, in the CDC5L overexpressed group, bcl-2 increased, while BAX and cleaved-caspase-3 levels decreased, with the opposite trends observed in the CDC5L silencing group (Fig. 3C). *In vivo* xenograft models further demonstrated that shCDC5L significantly reduced the volume and weight of subcutaneous tumors compared to the control (Fig. 3D-F). IHC analysis revealed increased E-cadherin expression and decreased Ki67, N-cadherin, and Vimentin levels in CDC5L-silenced cells (Fig. 3G-L). In summary, CDC5L silencing in GC cells promoted apoptosis and inhibited invasion, migration, and EMT.

### 3.4 CDC5L increased chemoresistance to oxaliplatin of GC cells

**Figure 4:**
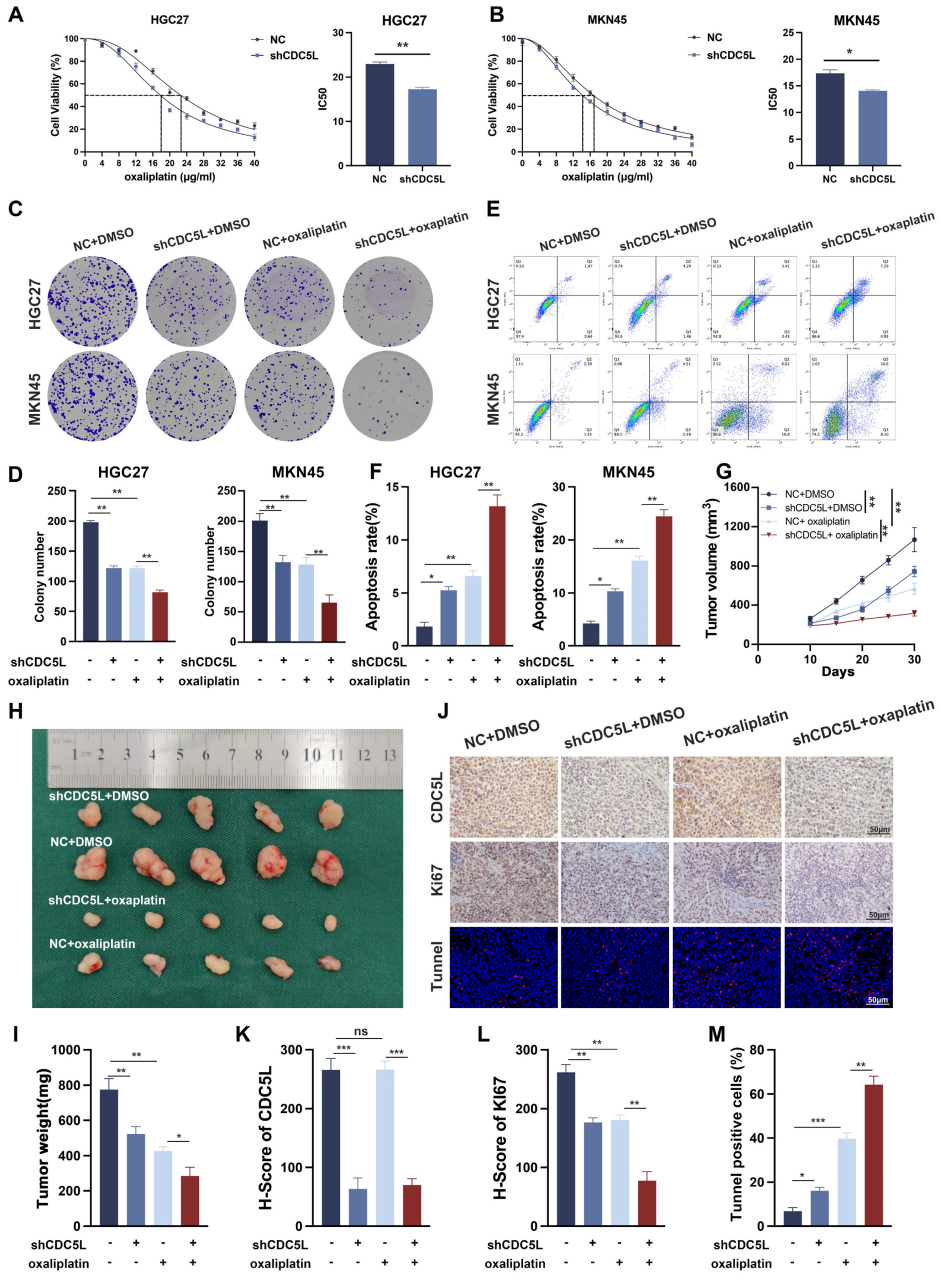
CDC5L increased chemoresistance to oxaliplatin of GC cells. **A, B.** CCK-8 was used for the evaluation of IC50 of GC cells exposed to oxaliplatin. **C, D.** Colony formation was used to detect the proliferation ability of GC cells in each group. **E, F.** Flow cytometry was used to detect the apoptosis of GC cells in each group. **G-I.** Subcutaneous tumorigenesis was performed in nude mice in each group and tumor volume and weight were measured. **J-M.** The expressions of CDC5L and Ki67 were detected by IHC and the percentage of tunnel was measured by IF in xenograft. CDDP concentrations: 8 μM in HGC27 for 24h and 1.5 μM in MKN45 for 24h. Error bars indicate SD. *P < 0.05, **P < 0.01, ***P < 0.001.

CDC5L has been implicated in the drug resistance of multiple myeloma 36. Our CCK-8 assay results demonstrated that the IC50 of GC cells decreased significantly upon CDC5L downregulation (Fig. 4A-B). Additionally, colony formation assays indicated that CDC5L suppression notably inhibited GC cell proliferation when exposed to oxaliplatin (Fig. 4C-D). Most notably, the apoptosis rate increased dramatically in the CDC5L-silenced group treated with oxaliplatin (Fig. 4E-F). Complementary *in vivo* studies corroborated these findings. Tumors in the shCDC5L + oxaliplatin group exhibited the lowest volume and weight among all tested groups (Fig. 4G-I). IHC analysis revealed a decrease in Ki67 expression and IF analysis showed a most significantly increase in TUNNEL staining in the shCDC5L + oxaliplatin group (Fig. 4J-M). To further confirm the role of CDC5L in chemoresistance in GC cells, HGC27-CDDP-resistant cells and MKN45-CDDP-resistant cells were generated. Colony formation and CCK-8 showed that the downregulation of CDC5L could weaken the chemoresistance in CDDP-resistant cells (Fig. S2A-B). Similar results were collected in the detection of apoptosis (Fig. S2C). Collectively, these *in vitro* and *in vivo* results confirm that CDC5L contributes to oxaliplatin resistance in GC cells.

### 3.5 Prp19 bound with CDC5L and modulated CDC5L expression in GC cells

**Figure 5:**
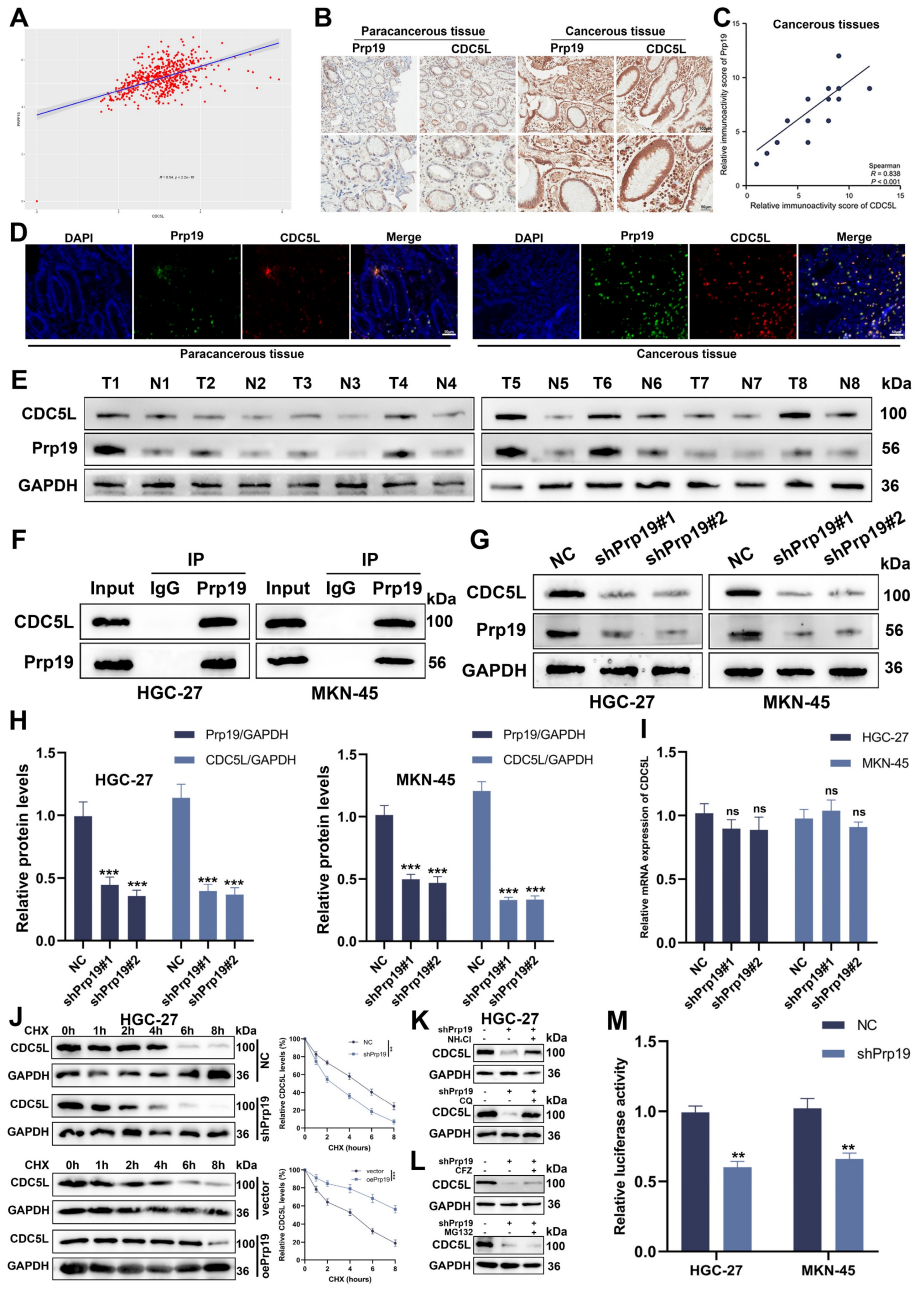
Prp19 bound with CDC5L and modulated CDC5L expression in GC cells. **A.** The positive correlation between Prp19 and CDC5L was analyzed based on the data from TCGA database. **B.** The IHC results of Prp19 and CDC5L in cancerous tissues and corresponding paracancerous tissues. **C.** The positive correlation between Prp19 and CDC5L was analyzed based on the IHC results of tumor tissues. **D.** Double immunofluorescence staining was used to detect Prp19 and CDC5L in cancerous tissues and corresponding paracancerous tissues. **E.** WB was used to detect the expression levels of CDC5L and Prp19 in tumor tissues and corresponding normal tissues. **F.** CO-IP was used to detect the interaction between CDC5L and Prp19. **G-I.** WB and qRT-PCR was used to detect the effects of shPrp19 on CDC5L expression. **J.** Detecting CDC5L protein levels in HGC27 cells under overexpression or knockdown of Prp19 subsequently subjected to the cycloheximide (CHX) chasing assay for indicated times. **K, L.** WB for CDC5L protein levels in HGC27 cells treated with NH_4_Cl (20 mM), chloroquine (100 mM), MG132 (20 mM) or Carfilzomib (100 nM) for 5 h before collection. **M.** Dual-luciferase reporter assay was used to measure the effects of Prp19 on CDC5L. Error bars indicate SD. *P < 0.05, **P < 0.01, ***P < 0.001.

Recent studies have reported a positive correlation between Prp19 and CDC5L^10^. Analysis from TCGA data shows a positive correlation between Prp19 and CDC5L (Fig. 5A). We further investigated this association in GC using IHC on the previously mentioned GC tissues. Our findings revealed colocalization of Prp19 with CDC5L, with both proteins exhibiting higher expression levels in GC tissues compared to corresponding paracancerous tissues (Fig. 5B, D). A positive correlation between Prp19 and CDC5L in tumor tissues was also confirmed (Fig. 5C), and similar results were observed in Western blot analyses (Fig. 5E). Co-IP experiments demonstrated the interaction between Prp19 and CDC5L (Fig. 5F), prompting further investigation into Prp19's regulatory role on CDC5L. Knockdown of Prp19 resulted in reduced CDC5L protein expression (Fig. 5G-H). To discern the mechanism, we evaluated CDC5L mRNA levels after Prp19 silencing and found negligible impact (Fig. 5I). To determine the effect of Prp19 on CDC5L stability, we used cycloheximide (CHX) to cease protein synthesis and monitored the degradation kinetics of CDC5L. As expected, Prp19 knockdown reduced the half-life of CDC5L, whereas Prp19 overexpression treatment delayed its degradation rate (Fig. 5J). There are two major mechanisms governing protein proteostasis: the autophagic lysosome system and the ubiquitin-proteasome pathway. Our investigation revealed that treatment with two lysosomal inhibitors, chloroquine (CQ) and NH_4_Cl reversed shPrp19-induced degradation of CDC5L in GC cells, whereas two proteasome inhibitors, carfilzomib (CFZ) and MG132 failed to support this effect (Fig. 5K-L), indicating that Prp19 may regulate CDC5L abundance via the lysosome pathway in GC cells. Additionally, luciferase assays assessing the transcriptional activity of CDC5L's 5'-UTR indicated decreased activity following shPrp19 transfection (Fig. 5M). In summary, Prp19 could bind with CDC5L and its silencing in GC cells leads to decreased CDC5L expression, primarily through lysosome-mediated degradation and suppression of mRNA translation.

### 3.6 Prp19 promoted malignant behaviors of GC via CDC5L

**Figure 6:**
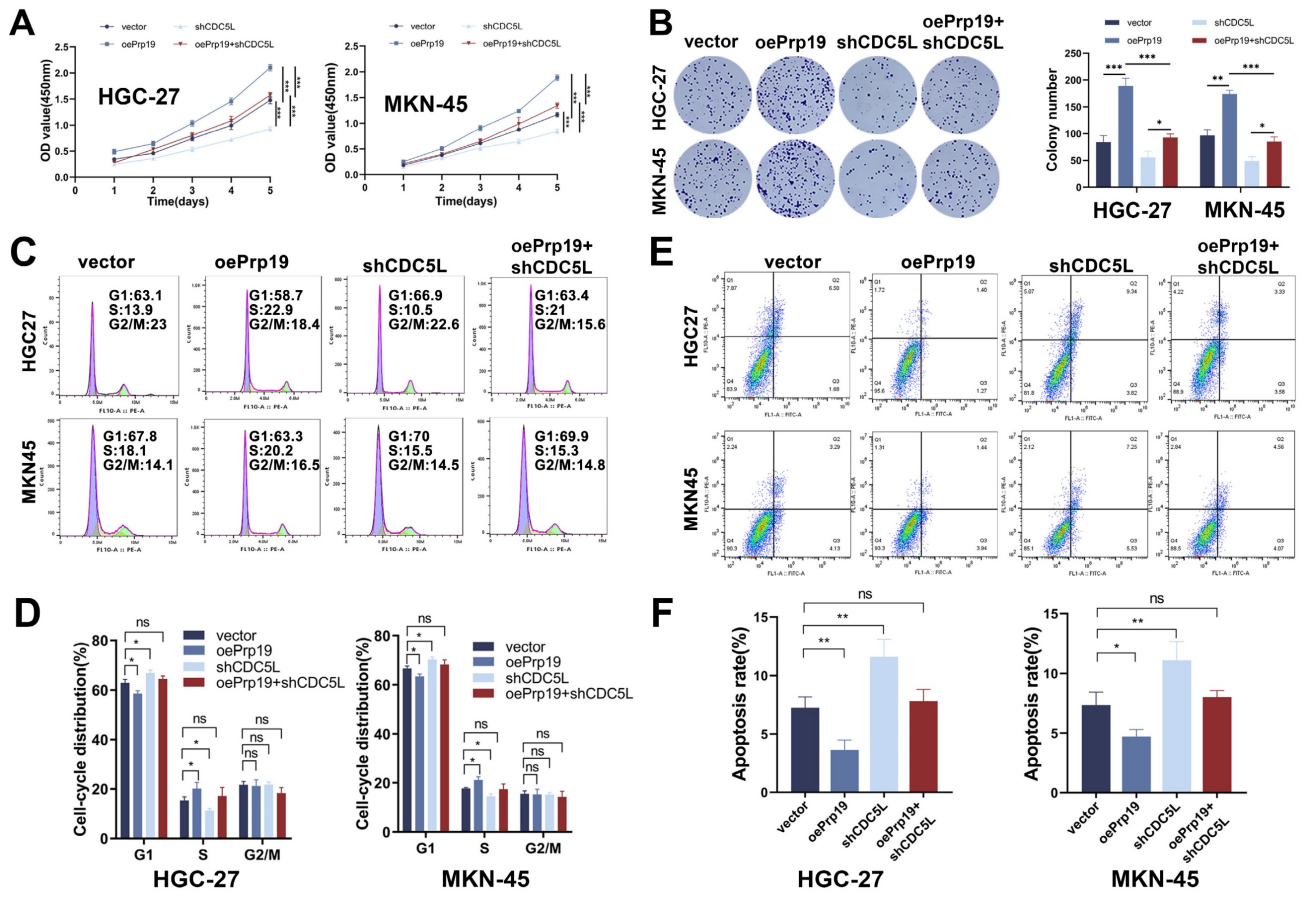
Prp19 promoted malignant behaviors of GC via CDC5L. **A, B.** CCK-8 and colony formation assays were used to detect the proliferation of GC cells in each group. **C, D.** Flow cytometry was used to detect the cell cycle of GC cells in each group. **E, F.** Flow cytometry was used to detect the apoptosis of GC cells in each group. Error bars indicate SD. *P < 0.05, **P < 0.01, ***P < 0.001.

We next explored the role of Prp19 in GC cell progression. The CCK-8 assay results indicated that Prp19 overexpression enhanced GC cell proliferation, an effect that could be mitigated by CDC5L silencing (Fig. 6A). This finding was corroborated by colony formation assays (Fig. 6B). Additionally, transwell and wound healing assays demonstrated that Prp19-mediated enhancement of GC cell migration and invasion could also be weakened by CDC5L knockdown (Fig. S3A-D). Moreover, Prp19 upregulation was associated with a decreased proportion of cells arrested in the G1 phase, an effect that could be rescued by the transfection with shCDC5L (Fig. 6C-D). Flow cytometry further revealed that shCDC5L negated the impact of oePrp19 on GC cell apoptosis (Fig. 6E-F). In summary, Prp19 promoted malignant behaviors in GC cells, a process that was dependent on CDC5L.

### 3.7 CDC5L interacted with MAPK1 and improved GC progression through MAPK1

**Figure 7:**
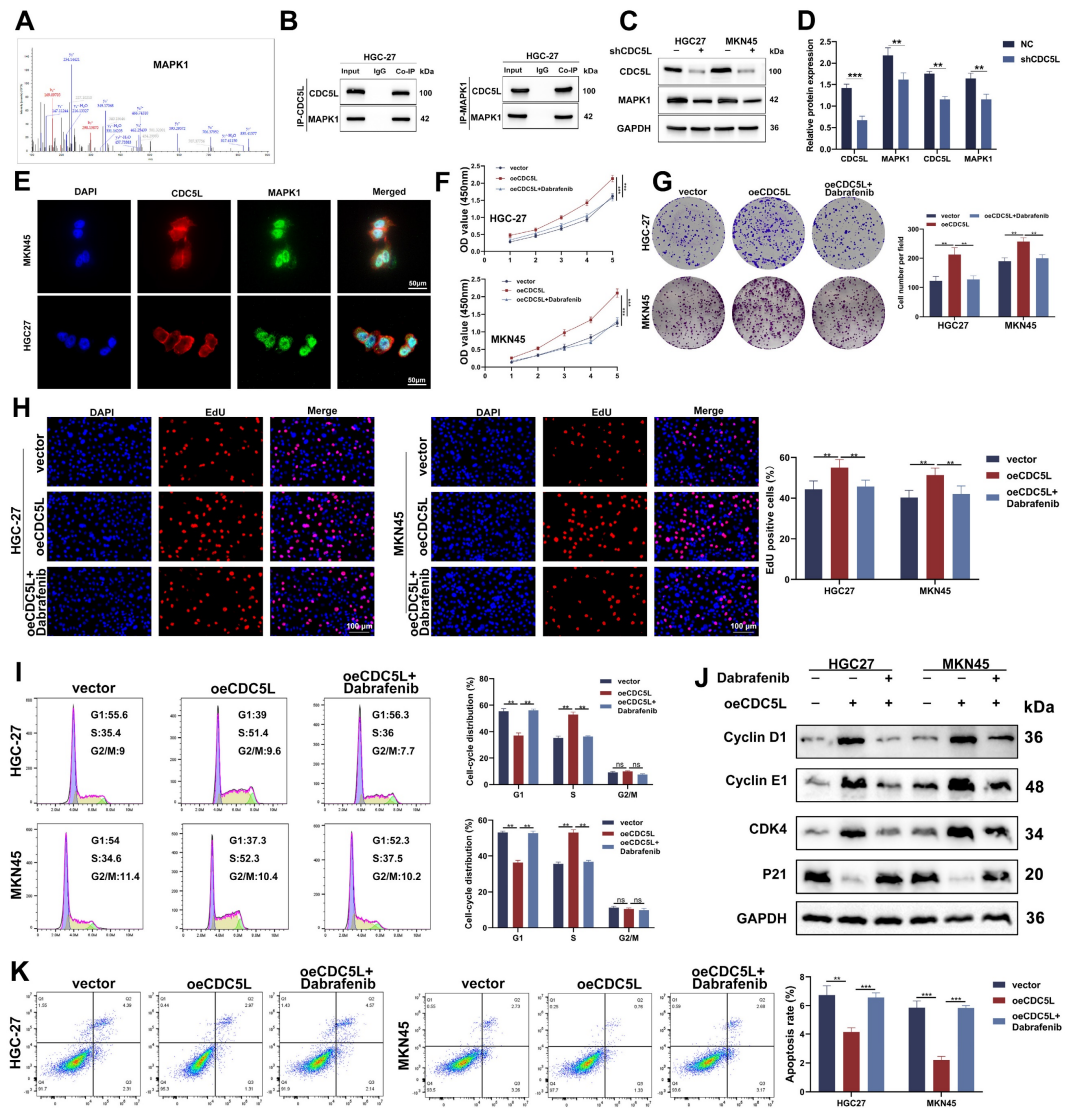
CDC5L interacted with MAPK1 and improved GC progression through MAPK1. **A.** CO-IP/mass spectrum assay was used to verify the interaction between CDC5L and MAPK1.** B.** WB revealed that CDC5L immunoprecipitated with anti-MAPK1 antibody, and MAPK1 immunoprecipitated with anti- CDC5L antibody. **C, D.** WB was used to detect the effects of shCDC5L on MAPK1 expression. **E.** IF was used to detect the localization of CDC5L and MAPK1. **F-H.** CCK-8, Colony formation and EdU assays were used to detect the proliferation of GC cells in each group. **I.** Flow cytometry was used to detect the cell cycle of GC cells in each group. **J.** WB was used for the detection of expression levels of cell cycle related proteins in each group. **K.** Flow cytometry was used to detect the apoptosis of GC cells in each group. Dabrafenib concentrations: 100 nM in HGC27 and 50 nM in MKN45 for 24h. Error bars indicate SD. *P < 0.05, **P < 0.01, ***P < 0.001.

To elucidate the downstream mechanism of CDC5L, CO-IP was employed to identify potential proteins which interacted with CDC5L. Mass spectrometry analysis suggested a strong interaction between CDC5L and MAPK1, indicated by high binding scores (Fig. 7A). Western blot analysis confirmed this interaction, showing that the anti-CDC5L antibody could immunoprecipitate MAPK1 and vice versa (Fig. 7B). Notably, CDC5L knockdown resulted in decreased protein levels of MAPK1 (Fig. 7C-D), and IF assays revealed their colocalization in the nucleus (Fig. 7E). Dabrafenib, inhibitor of MAPK pathway, was used to explore the effects of CDC5L on the activation of MAPK pathway. Functional assays, including CCK-8, colony formation, and EdU incorporation, demonstrated that MAPK1 silencing impeded GC cell proliferation, which could be rescued by CDC5L overexpression (Fig. 7F-H, Fig. S4A-C). Additionally, CDC5L overexpression reduced the number of cells arrested in the G1 phase, which could be counteracted by MAPK1 silencing (Fig. 7I, Fig. S4D). These observations align with Western blot analyses of cell cycle-related proteins (Fig. 7J, Fig. S4E). Furthermore, flow cytometry of apoptosis indicated that MAPK1 knockdown mitigated the protective effects of CDC5L on GC cells (Fig. 7K, Fig. S4F). Organoid model revealed that MAPK1 silencing inhibited the growth of organoid, which could be reversed by CDC5L overexpression (Fig. S4G). Collectively, these findings suggest that CDC5L modulates GC progression through its interaction with MAPK1.

### 3.8 CDC5L enhanced HR via MAPK pathway activation

**Figure 8:**
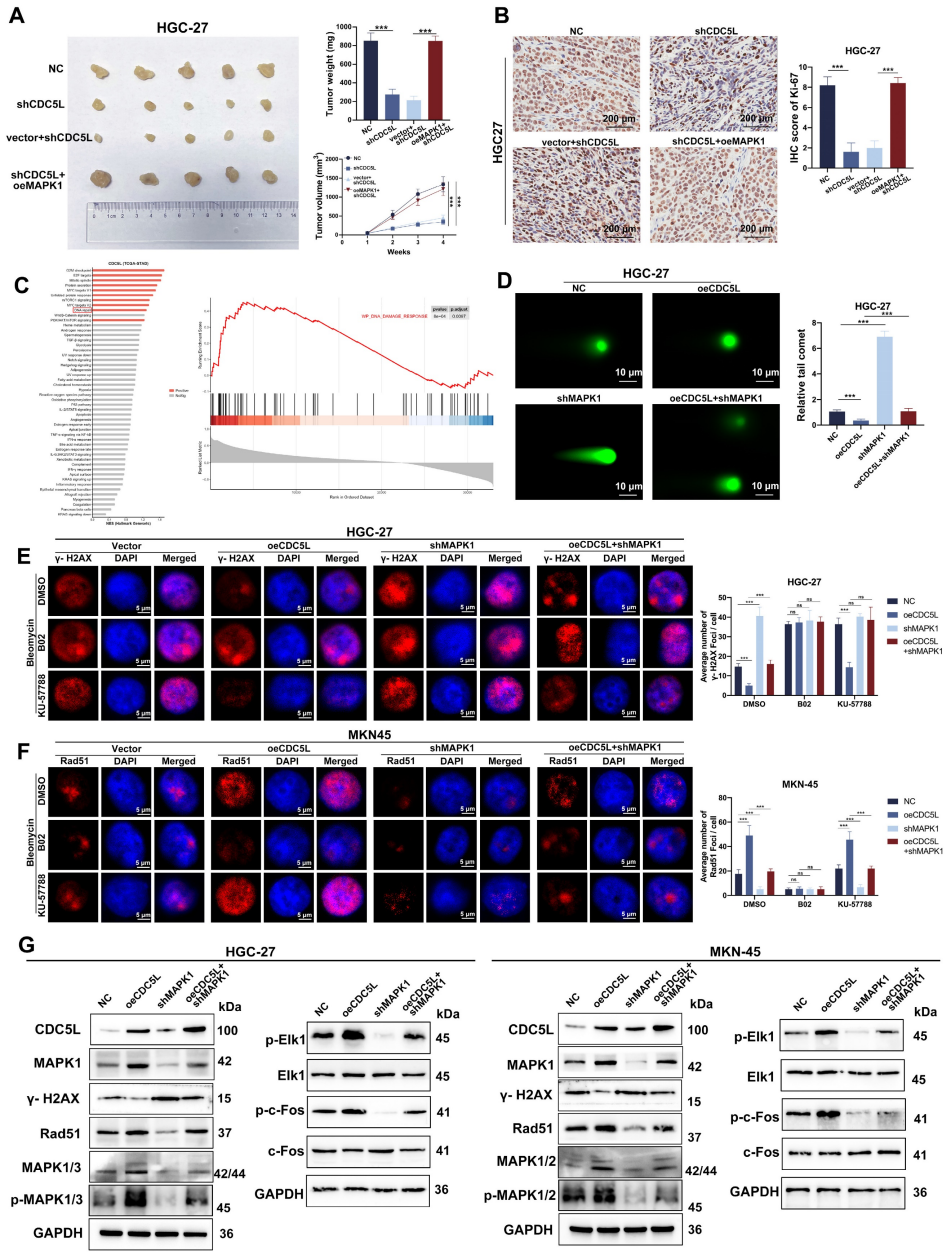
CDC5L enhanced HR via MAPK pathway activation. **A.** Subcutaneous tumorigenesis was performed in nude mice in each group and tumor volume and weight were measured. **B.** The expressions of Ki67 in each group were detected by IHC in xenograft. **C.** Bioinformatic tools were used to verify the positive correlation between CDC5L and DNA damage response. **D.** Representative images of neutral comet assays performed of GC cells in each group. **E, F.** Representative images of γ-H2A.X and Rad51 foci of GC cells in each group. **G.** WB was used to detect the expressions of MAPK pathway related proteins in each group. Error bars indicate SD. *P < 0.05, **P < 0.01, ***P < 0.001.

We further explored MAPK1's role *in vivo* using nude mice, which were divided into four groups: NC (negative control), shCDC5L, shCDC5L+vector, and shCDC5L+oeMAPK1. Tumors in the shCDC5L group were significantly smaller in both volume and weight compared to the NC and shCDC5L+oeMAPK1 groups (Fig. 8A). This reduction paralleled a decrease in Ki-67 expression, a marker of proliferation (Fig. 8B). Gene Set Enrichment Analysis (GSEA) indicated a positive correlation between CDC5L expression and DNA damage response pathways (Fig. 8C).

Comet assays demonstrated that CDC5L overexpression led to a reduction in comet tail length, an effect reversed by MAPK1 silencing (Fig. 8D). IF studies revealed that oeCDC5L-induced reduction of γ-H2AX foci, a marker of DNA damage, was counteracted by shMAPK1. Additionally, B02, an inhibitor of HR, diminished the influence of CDC5L and MAPK1 on homologous recombination (HR) repair. However, KU-57788, an inhibitor of NHEJ, had no influence on the DSBR promoted by MAPK1 (Fig. 8E). Furthermore, shMAPK1 abrogated the enhancement of Rad51 foci formation, essential for DNA damage repair, caused by CDC5L overexpression (Fig. 8F). Subsequently, we delved into the molecular mechanism underlying the promotion of HR by CDC5L and MAPK1. The MAPK pathway is known to play a crucial role in DNA damage response, with MAPK1 being a key activator 38. Western blot analysis revealed that CDC5L overexpression significantly upregulated MAPK1/3, phosphorylated MAPK1/3 (p-MAPK1/3), and downstream effectors of MAPK1 including p-ELK1, p-c-Fos. Such elevation was reversed by shMAPK1 (Fig. 8G). Additionally, CDC5L overexpression led to decreased γ-H2A and increased Rad51 expression. To Thus, we concluded that CDC5L augments HR through MAPK pathway activation.

To further explore the potential of regulating CDC5L in homologous recombination repair, Olaparib was used. Olaparib is known to be the inhibitor of PARP. Xenograft model revealed that knockdown of CDC5L and PARP inhibitors had a synergistic effect in inhibiting tumor growth (Fig. S4H).

**Figure 9:**
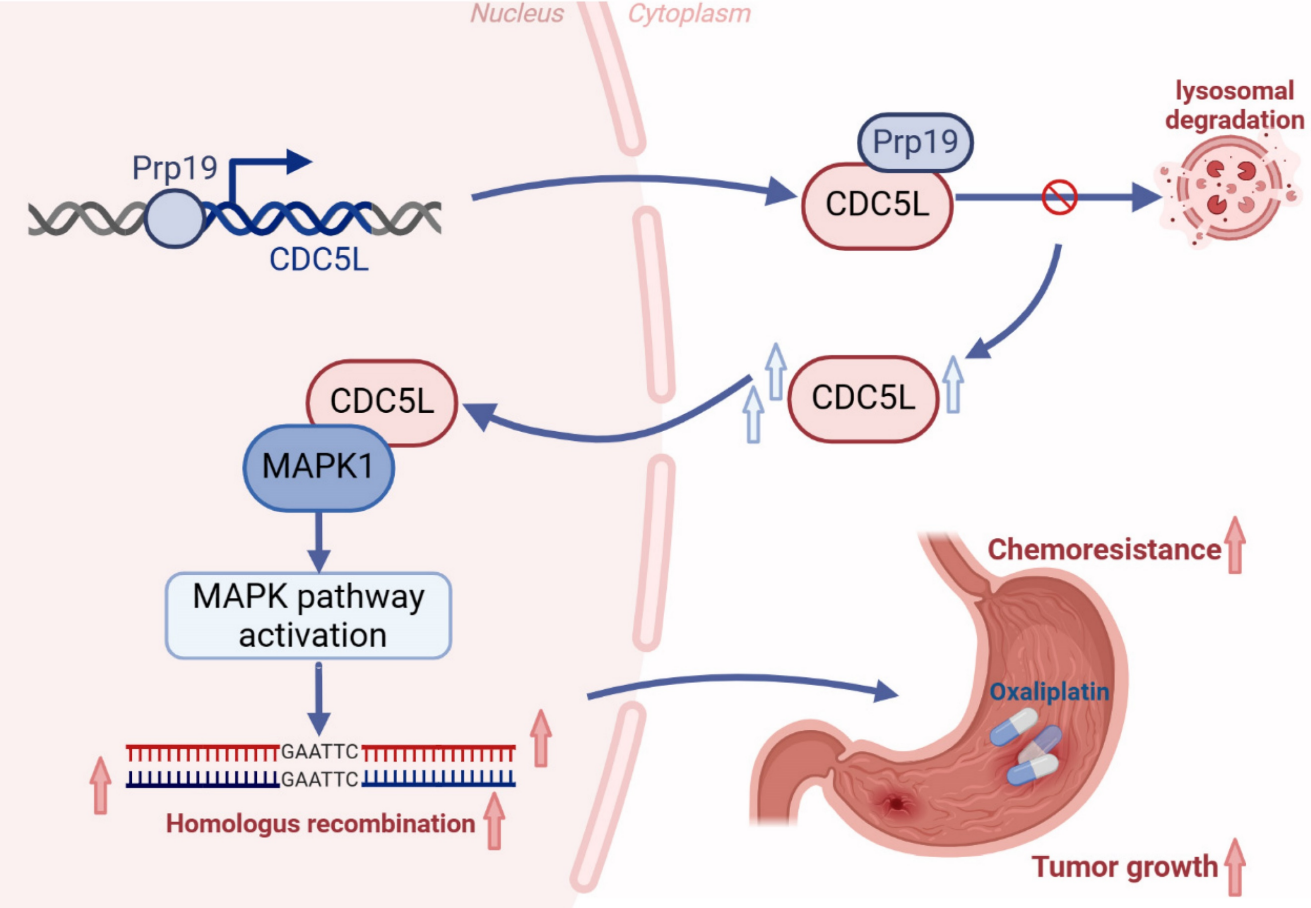
Schedule diagram of the results in this work. In summary, our study confirms that Prp19 upregulates CDC5L expression, which binds to MAPK1, thereby promoting GC progression via the MAPK pathway-mediated homologous recombination.

Taken together, our study confirms that Prp19 upregulates CDC5L expression, which binds to MAPK1, thereby promoting GC progression via the MAPK pathway-mediated homologous recombination (Fig. 9).

## 4. Discussion

As the fourth leading cause of cancer-related deaths globally, GC has a poor prognosis, partly due to its complex molecular mechanisms 1. CDC5L, known to be involved in various cancers, including non-small cell lung cancer, hepatocellular cancer, and colorectal cancer 11-14, was observed in our study to be associated with larger tumor volume, advanced T and N stages, and advanced pathological grades in GC. Patients with lower CDC5L expression had better prognoses compared to those with higher expression. We found that elevated CDC5L levels in GC cells enhanced proliferation, invasion, and migration while reducing apoptosis and inhibiting cell cycle arrest in the G1 phase. Furthermore, recent studies have suggested the association between cell cycle-related proteins and platinum resistance^39^. Huang *et al.* found CDC5L promoted the cell cycle, proliferation, and cell adhesion-mediated drug resistance of multiple myeloma cells 13. Consistent with this, our results indicate that silencing CDC5L in GC cells reduced chemoresistance to oxaliplatin.

Prp19, a key component of the Prp19-CDC5L complex, plays crucial roles in pre-mRNA splicing and DNA damage repair^5^, ^40^. Shen *et al.* found that Prp19 was involved in tumor formation and progression^41^. Our study revealed that Prp19 was positively correlated with CDC5L. Through Co-IP, we discovered Prp19's interaction with CDC5L. Prp19 could bind with CDC5L and Prp19 silencing reduced CDC5L expression via promoting lysosome-mediated degradation and suppressing mRNA transcription in GC cells. Additionally, Prp19 upregulation led to a decreased proportion of cells arrested in the G1 phase, reversible by shCDC5L transfection, indicating Prp19's promotion of GC malignancy is CDC5L-dependent.

We further investigated CDC5L's molecular mechanisms in regulating malignant behaviors of GC. By means of MS and CO-IP, we first identified CDC5L's interaction with MAPK1, a key component in activating the MAPK pathway 33, which is implicated in various malignant biological behaviors of tumors, including drug resistance and DNA damage repair 36. In our study, MAPK1 silencing inhibited proliferation of GC cells, which could be rescued by overexpressing CDC5L. Besides, overexpressed CDC5L contributed to the decrease of GC cells arrested in G1 phase, which could be counteracted by MAPK1 silencing. These results were also supported by western blot analysis of cell cycle proteins and flow cytometry of apoptosis, showing that MAPK1 knockdown attenuated CDC5L's protective effects on GC cells.

Oxaliplatin-based chemotherapy is the recommended treatment for advanced GC, but overcoming chemoresistance remains a critical challenge to improve patient prognosis 24. Oxaliplatin is able to form platinum-DNA adducts to cause DSBs, contributing to the death of rapidly growing cells 27. Accumulating evidence revealed that HR plays a vital role in modulating chemoresistance. Our study demonstrated CDC5L's positive association with DNA damage response, enhancing HR through MAPK pathway as evidenced by the downregulation of γ-H2A, upregulation of Ras51, and activation of the MAPK pathway related molecules.

In conclusion, our study is the first to demonstrate that the Prp19-CDC5L complex promotes HR and thus inhibits chemo-sensitivity in GC via MAPK pathway activation. CDC5L emerges as a potential therapeutic and prognostic target in GC, warranting further investigation for targeted therapies.

## Supplementary Material

Supplementary figures and tables.

## Figures and Tables

**Table 1:** Correlations between CDC5L expression and clinicopathologic features of GC patients

Characteristics	CDC5L		P value
	high	low	
n	40	40	
Age			
<60 years	11 (13.8%)	8 (10%)	0.431
≥60 years	29 (36.2%)	32 (40%)	
Gender			
Male	30 (37.5%)	29 (36.2%)	0.799
Female	10 (12.5%)	11 (13.8%)	
Tumor size			
<3cm	4 (5%)	15 (18.8%)	0.004**
≥3cm	36 (45%)	25 (31.2%)	
Tumor invasion			
T1+T2	3 (3.8%)	16 (20%)	< 0.001***
T3+T4	37 (46.2%)	24 (30%)	
Lymph node metastasis			
Negative	11 (13.8%)	23 (28.7%)	0.007*
Positive	29 (36.2%)	17 (21.2%)	
Tumor differentiation			
Well and moderate	12 (15%)	17 (21.2%)	0.245
Poor	28 (35%)	23 (28.7%)	
TNM stage			
Ⅰ-Ⅱ	6 (7.5%)	24 (30%)	< 0.001***
Ⅲ	34 (42.5%)	16 (20%)	

**Table 2:** Results of variate analysis for overall survival in GC tissue.

Characteristics	Total (N)	Univariate analysis			Multivariate analysis	
		Hazard ratio (95% CI)	P value		Hazard ratio (95% CI)	P value
CDC5L	80					
low	40	Reference			Reference	
high	40	0.461 (0.248 - 0.857)	**0.014**		0.461 (0.248 - 0.857)	**0.014**
Age	80					
<60 years	19	Reference				
≥60 years	61	0.869 (0.428 - 1.763)	0.697			
Gender	80					
Male	59	Reference				
Female	21	1.717 (0.796 - 3.703)	0.168			
Tumor size	80					
<3cm	19	Reference				
≥3cm	61	1.164 (0.586 - 2.309)	0.665			
Tumor invasion	80					
T1+T2	19	Reference				
T3+T4	61	0.723 (0.347 - 1.508)	0.387			
Lymph node metastasis	80					
Negative	34	Reference				
Positive	46	1.163 (0.638 - 2.118)	0.622			
Tumor differentiation	80					
Well and moderate	29	Reference				
Poor	51	1.009 (0.544 - 1.874)	0.977			
TNM stage	80					
Ⅰ-Ⅱ	30	Reference				
Ⅲ	50	0.971 (0.523 - 1.803)	0.927			

## References

[1] Bray F, Laversanne M, Sung H, Ferlay J, Siegel RL, Soerjomataram I (2024). Global cancer statistics 2022: GLOBOCAN estimates of incidence and mortality worldwide for 36 cancers in 185 countries. CA Cancer J Clin.

[2] Bae JS, Chang W, Kim SH, Choi Y, Kong SH, Lee HJ (2022). Development of a predictive model for extragastric recurrence after curative resection for early gastric cancer. Gastric Cancer.

[3] Li S, Bao J, Li X, Yang Q, Xu J, Chen S (2023). Multicenter phase I dose escalation and expansion study of pyrotinib in combination with camrelizumab and chemotherapy as first-line treatment for HER2-positive advanced gastric and gastroesophageal junction adenocarcinoma. EClinicalMedicine.

[4] Mu R, Wang YB, Wu M, Yang Y, Song W, Li T (2014). Depletion of pre-mRNA splicing factor Cdc5L inhibits mitotic progression and triggers mitotic catastrophe. Cell Death Dis.

[5] Wahl MC, Will CL, Luhrmann R (2009). The spliceosome: design principles of a dynamic RNP machine. Cell.

[6] Grote M, Wolf E, Will CL, Lemm I, Agafonov DE, Schomburg A (2010). Molecular architecture of the human Prp19/CDC5L complex. Mol Cell Biol.

[7] Zhang N, Kaur R, Akhter S, Legerski RJ (2009). Cdc5L interacts with ATR and is required for the S-phase cell-cycle checkpoint. EMBO Rep.

[8] Ajuh P, Lamond AI (2003). Identification of peptide inhibitors of pre-mRNA splicing derived from the essential interaction domains of CDC5L and PLRG1. Nucleic Acids Res.

[9] Qiu H, Zhang X, Ni W, Shi W, Fan H, Xu J (2016). Expression and Clinical Role of Cdc5L as a Novel Cell Cycle Protein in Hepatocellular Carcinoma. Dig Dis Sci.

[10] Huang R, Xue R, Qu D, Yin J, Shen XZ (2017). Prp19 Arrests Cell Cycle via Cdc5L in Hepatocellular Carcinoma Cells. Int J Mol Sci.

[11] Chen G, Wei RS, Ma J, Li XH, Feng L, Yu JR (2023). FOXA1 prolongs S phase and promotes cancer progression in non-small cell lung cancer through upregulation of CDC5L and activation of the ERK1/2 and JAK2 pathways. Kaohsiung J Med Sci.

[12] Wang K, Li B, Fan P, Ren X, Jiang H (2021). Downregulation of DEAD-box helicase 21 (DDX21) inhibits proliferation, cell cycle, and tumor growth in colorectal cancer via targeting cell division cycle 5-like (CDC5L). Bioengineered.

[13] Liu H, Guo D, Sha Y, Zhang C, Jiang Y, Hong L (2020). ANXA7 promotes the cell cycle, proliferation and cell adhesion-mediated drug resistance of multiple myeloma cells by up-regulating CDC5L. Aging (Albany NY).

[14] Zhang C, Li Y, Zhao W, Liu G, Yang Q (2020). Circ-PGAM1 promotes malignant progression of epithelial ovarian cancer through regulation of the miR-542-3p/CDC5L/PEAK1 pathway. Cancer Med.

[15] Jackson SP, Bartek J (2009). The DNA-damage response in human biology and disease. Nature.

[16] Waterman DP, Haber JE, Smolka MB (2020). Checkpoint Responses to DNA Double-Strand Breaks. Annu Rev Biochem.

[17] Geng L, Zhu M, Luo D, Chen H, Li B, Lao Y (2024). TKT-PARP1 axis induces radioresistance by promoting DNA double-strand break repair in hepatocellular carcinoma. Oncogene.

[18] Altwerger G, Ghazarian M, Glazer PM (2023). Harnessing the effects of hypoxia-like inhibition on homology-directed DNA repair. Semin Cancer Biol.

[19] Sekhar KR, Freeman ML (2023). Nucleophosmin Plays a Role in Repairing DNA Damage and Is a Target for Cancer Treatment. Cancer Res.

[20] Ghezraoui H, Piganeau M, Renouf B, Renaud JB, Sallmyr A, Ruis B (2014). Chromosomal translocations in human cells are generated by canonical nonhomologous end-joining. Mol Cell.

[21] Laurini E, Marson D, Fermeglia A, Aulic S, Fermeglia M, Pricl S (2020). Role of Rad51 and DNA repair in cancer: A molecular perspective. Pharmacol Ther.

[22] Brinkman JA, Liu Y, Kron SJ (2021). Small-molecule drug repurposing to target DNA damage repair and response pathways. Semin Cancer Biol.

[23] Marin JJ, Al-Abdulla R, Lozano E, Briz O, Bujanda L, Banales JM (2016). Mechanisms of Resistance to Chemotherapy in Gastric Cancer. Anticancer Agents Med Chem.

[24] Whitehurst AW (2014). Cause and consequence of cancer/testis antigen activation in cancer. Annu Rev Pharmacol Toxicol.

[25] Chen Z, Xu P, Wang X, Li Y, Yang J, Xia Y (2023). MSC-NPRA loop drives fatty acid oxidation to promote stemness and chemoresistance of gastric cancer. Cancer Lett.

[26] Rocha CRR, Silva MM, Quinet A, Cabral-Neto JB, Menck CFM (2018). DNA repair pathways and cisplatin resistance: an intimate relationship. Clinics (Sao Paulo).

[27] Galluzzi L, Senovilla L, Vitale I, Michels J, Martins I, Kepp O (2012). Molecular mechanisms of cisplatin resistance. Oncogene.

[28] Yang J, Xu P, Chen Z, Zhang X, Xia Y, Fang L (2023). N6-methyadenosine modified SUV39H2 regulates homologous recombination through epigenetic repression of DUSP6 in gastric cancer. Cancer Lett.

[29] Motegi A, Masutani M, Yoshioka KI, Bessho T (2019). Aberrations in DNA repair pathways in cancer and therapeutic significances. Semin Cancer Biol.

[30] Brown JS, O'Carrigan B, Jackson SP, Yap TA (2017). Targeting DNA Repair in Cancer: Beyond PARP Inhibitors. Cancer Discov.

[31] Yang SH, Sharrocks AD, Whitmarsh AJ (2013). MAP kinase signalling cascades and transcriptional regulation. Gene.

[32] Jiang T, Xia Y, Lv J, Li B, Li Y, Wang S (2021). A novel protein encoded by circMAPK1 inhibits progression of gastric cancer by suppressing activation of MAPK signaling. Mol Cancer.

[33] Burotto M, Chiou VL, Lee JM, Kohn EC (2014). The MAPK pathway across different malignancies: a new perspective. Cancer.

[34] Vashisht Gopal YN, Gammon S, Prasad R, Knighton B, Pisaneschi F, Roszik J (2019). A Novel Mitochondrial Inhibitor Blocks MAPK Pathway and Overcomes MAPK Inhibitor Resistance in Melanoma. Clin Cancer Res.

[35] Ma Y, Wang L, Neitzel LR, Loganathan SN, Tang N, Qin L (2017). The MAPK Pathway Regulates Intrinsic Resistance to BET Inhibitors in Colorectal Cancer. Clin Cancer Res.

[36] Li W, Melton DW (2012). Cisplatin regulates the MAPK kinase pathway to induce increased expression of DNA repair gene ERCC1 and increase melanoma chemoresistance. Oncogene.

[37] Li S, Fong KW, Gritsina G, Zhang A, Zhao JC, Kim J (2019). Activation of MAPK Signaling by CXCR7 Leads to Enzalutamide Resistance in Prostate Cancer. Cancer Res.

[38] Jeayeng S, Wongkajornsilp A, Slominski AT, Jirawatnotai S, Sampattavanich S, Panich U (2017). Nrf2 in keratinocytes modulates UVB-induced DNA damage and apoptosis in melanocytes through MAPK signaling. Free Radic Biol Med.

[39] Shah MA, Schwartz GK (2001). Cell cycle-mediated drug resistance: an emerging concept in cancer therapy. Clin Cancer Res.

[40] Ohi MD, Vander Kooi CW, Rosenberg JA, Ren L, Hirsch JP, Chazin WJ (2005). Structural and functional analysis of essential pre-mRNA splicing factor Prp19p. Mol Cell Biol.

[41] Yin J, Zhu JM, Shen XZ (2012). New insights into pre-mRNA processing factor 19: A multi-faceted protein in humans. Biol Cell.

