# Supplementary material for: Prp19/CDC5L promotes gastric cancer via activation of the MAPK pathway-mediated homologous recombination
Source: Int J Biol Sci. 2025 Jan 27;21(4):1603–18. doi: 10.7150/ijbs.101962 (PMC11844288; doi:10.7150/ijbs.101962)
Supplement: Supplementary file 1 — Supplementary figures and tables. [file ijbsv21p1603s1.pdf]

Supplementary Figure. 1

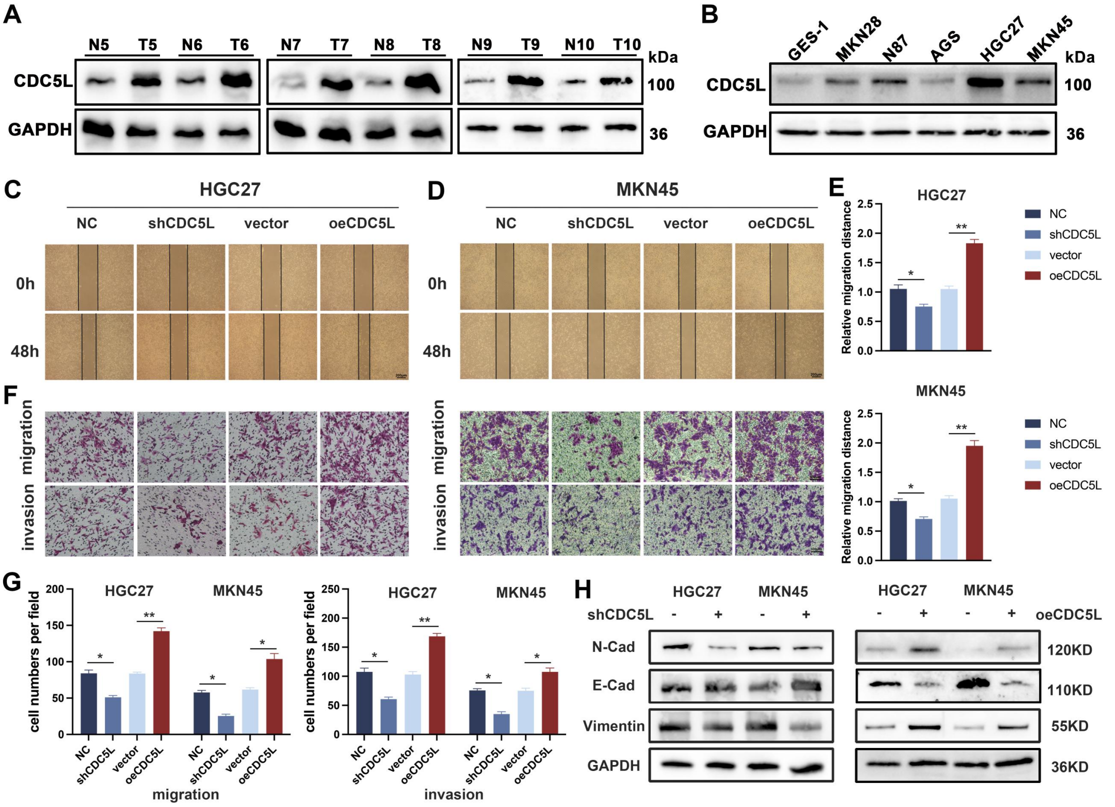

A. Protein expressions of CDC5L in GC tissues and adjacent normal tissues were analyzed by WB. **B.** WB was utilized to detect the expression levels of CDC5L in GC cells. **C-F.** Cell migration and invasion abilities were detected by wound healing and transwell assays after silencing and overexpression of CDC5L in GC cells. **G.** WB was used for the detection of expression levels of EMT related proteins after silencing and overexpression of CDC5L. Error bars indicate SD. *P < 0.05, **P < 0.01, ***P < 0.001.

Supplementary Figure. 2

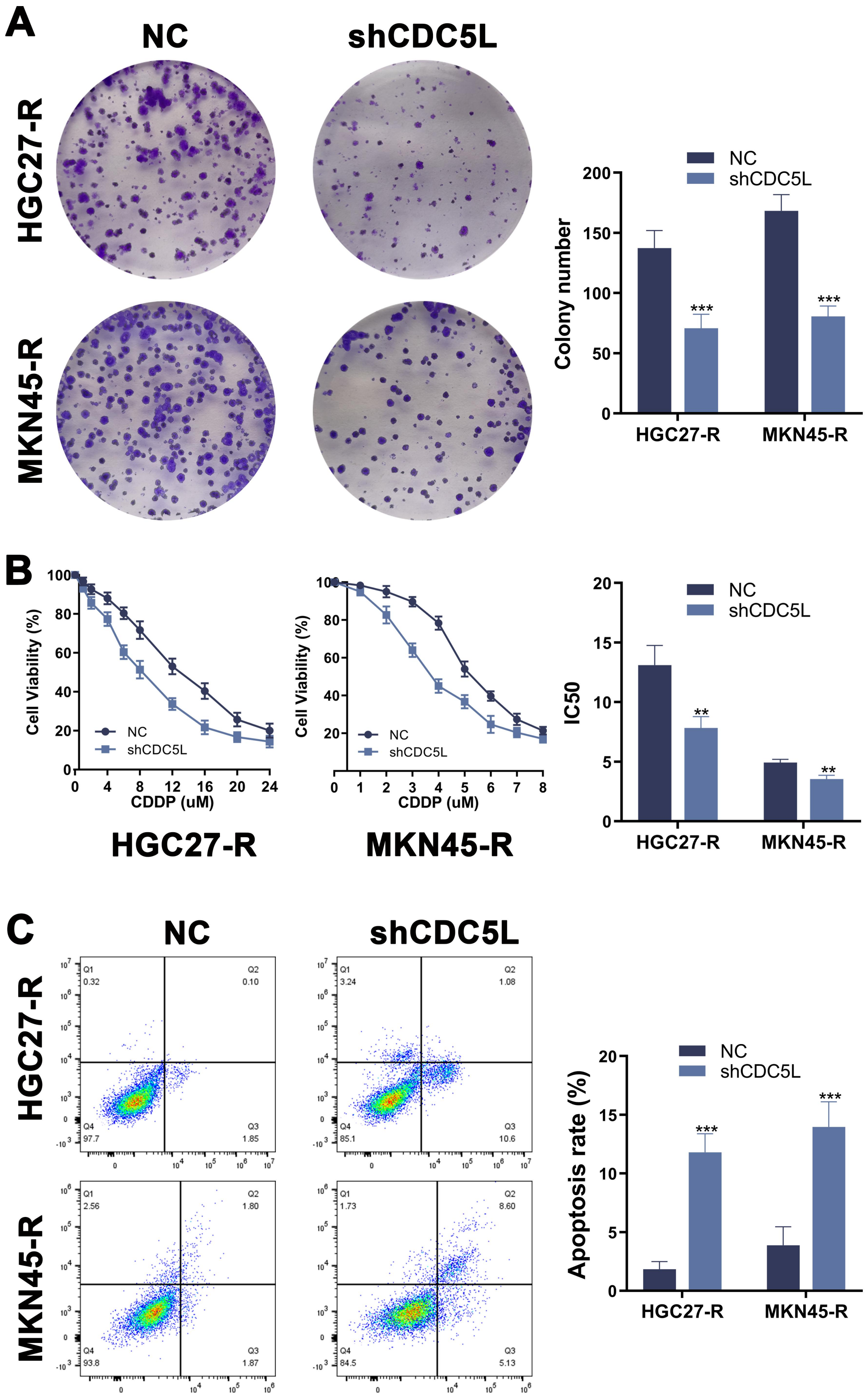

A. Representative graphs of colony formation of CDDP-resistant GC cells treated with CDDP

after downregulating CDC5L. **B.** CCK-8 assays were used to detect the proliferation of CDDP-resistant GC cells treated with CDDP after downregulating CDC5L. **C.** Flow cytometry was used to detect the apoptosis of CDDP-resistant GC cells treated with CDDP after downregulating CDC5L. CDDP concentrations: 12 μM in HGC27-R for 24h and 3 μM in MKN45-R for 24h. Error bars indicate SD. *P < 0.05, **P < 0.01, ***P < 0.001.

Supplementary Figure. 3

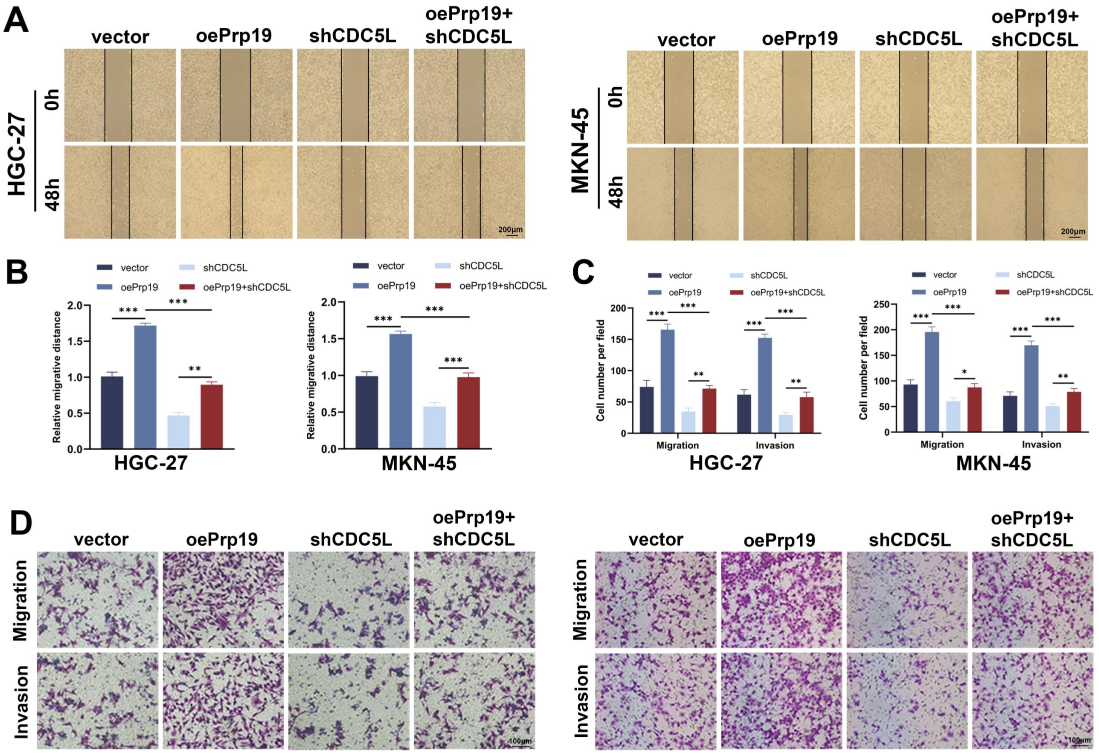

A, B. Wound healing assays were used to measure the migration of GC cells in each group. **C, D.** Transwell assays were used to measure the migration and invasion of GC cells in each group. Error bars indicate SD. *P < 0.05, **P < 0.01, ***P < 0.001.

Supplementary Figure. 4

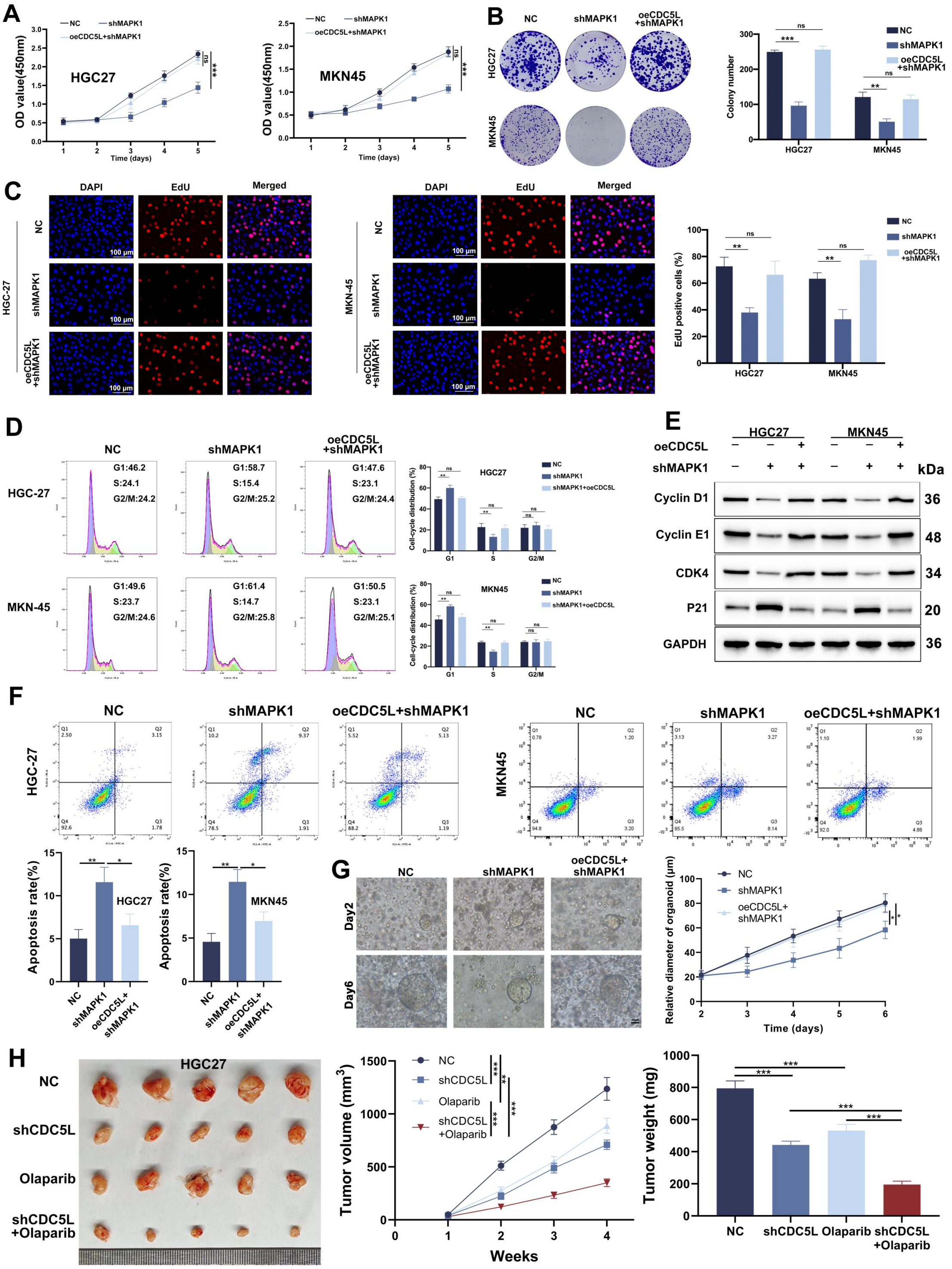

A-C. , CCK-8, Colony formation and EdU assays were used to detect the proliferation of GC cells in each group. **D.** Flow cytometry was used to detect the cell cycle of GC cells in each group. **E.** WB was used for the detection of expression levels of cell cycle related proteins in each group. **F.** Flow cytometry was used to detect the apoptosis of GC cells in each group. **G.** Organoid was measured to examine the effects of CDC5L and MAPK1 on GC. Error bars indicate SD. **H.**

Subcutaneous tumorigenesis was performed in nude mice in each group and tumor volume and weight were measured. Olaparib (50mg/kg) was administered daily. Error bars indicate SD. *P < 0.05, **P < 0.01, ***P < 0.001.

Supplementary Table. 1 Sequences of primers used in this study

Primer sequence	
GAPDH	Forward: 5’-GTCAAGGCTGAGAACGGGAA-3’<br>Reverse: 5’-AAATGAGCCCCAGCCTTCTC-3’
CDC5L	Forward: 5′-TCTCTGAAGCTCCTCTCGGC-3′<br>Reverse: 5′-CATCCTCGGTATTCCTCCATACG-3′

Supplementary Table. 2 Antibodies used in this study

Western blot

Primary antibody		
CDC5L	Abcam	Ab314000
Cyclin D1	Proteintech	26939-1-AP
Cyclin E1	Proteintech	11554-1-AP
CDK4	Proteintech	11026-1-AP
P21	Proteintech	10355-1-AP
Bcl-2	Abcam	Ab32124
BAX	Abcam	Ab182733
Cleaved-caspase3	Abcam	Ab2302
E-Cadherin	Abcam	Ab40772
N-Cadherin	Abcam	Ab76011
Vimentin	Abcam	Ab92547
Ki67	Abcam	Ab15580
Prp19	Abcam	Ab126776
γ-H2A.X	Abcam	Ab81299
Rad51	Abcam	Ab133534
MAPK1	Abcam	Ab32527
MAPK1/3	Abcam	Ab184699
p-MAPK1/3	Abcam	Ab201015
p-EIk1	Abcam	Ab218133
p-c-FOS	Abcam	Ab308128
p-c-JUN	Abcam	Ab32385
p-RSK1	Abcam	Ab32114
GAPDH	Abcam	Ab8245
Secondary antibody		
Anti-rabbit IgG	Cell signaling Technology	#7074
Anti-mouse IgG	Cell signaling Technology	#7076

IHC and IF

Primary antibody		
CDC5L	Abcam	Ab314000
E-Cadherin	Abcam	Ab40772
N-Cadherin	Abcam	Ab76011
Vimentin	Abcam	Ab92547

Ki67	Abcam	Ab15580
Prp19	Abcam	Ab126776
γ-H2A.X	Abcam	Ab81299
Rad51	Abcam	Ab133534
Secondary antibody		
Goat Anti-Mouse IgG H&L (Alexa Fluor® 647)	Abcam	Ab150115
Goat Anti-Rabbit IgG H&L (Alexa Fluor® 594)	Abcam	Ab150080
